# The roles of short-chain fatty acids derived from colonic bacteria fermentation of non-digestible carbohydrates and exogenous forms in ameliorating intestinal mucosal immunity of young ruminants

**DOI:** 10.3389/fimmu.2023.1291846

**Published:** 2023-12-11

**Authors:** Zhiyuan He, Hong Dong

**Affiliations:** Beijing Traditional Chinese Veterinary Engineering Center and Beijing Key Laboratory of Traditional Chinese Veterinary Medicine, Beijing University of Agriculture, Beijing, China

**Keywords:** dairy calves, gut microbiota, intestinal mucosal immunity, non-digestible carbohydrates, short-chain fatty acids

## Abstract

Short-chain fatty acids (SCFA) are a class of organic fatty acids that consist of 1 to 6 carbons in length. They are primary end-products which arise from non-digestible carbohydrates (NDC) fermentation of colonic bacteria. They are the fundamental energy sources for post-weaning ruminants. SCFA represent the major carbon flux of diet through the gut microbiota to the host. They also play a vital role in regulating cell expansion and gene expression of the gastrointestinal tract (GIT). Recently, remarkable progresses have been made in understanding the immunomodulatory effects of SCFA and their interactions with the host. The processes involved in this study encompassed inflammasome activation, proliferation of lymphocytes, and maturation of intestinal mucosal immunity maturation. It is important to note that the establishment and maturation of intestinal mucosal immune system are intricately connected to the barrier function of intestinal epithelial cells (IEC) and the homeostasis of gut microbiota. Thus, insights into the role of SCFA in enteric mucosal immunoreaction of calves will enhance our understanding of their various regulatory functions. This review aims to analyze recent evidence on the role of SCFA as essential signaling molecules between gut microbiota and animal health. Additionally, we provide a summary of current literature on SCFA in intestinal mucosal immune responses of dairy calves.

## Introduction

Neonatal calves are primarily protected by their innate immune system, as essential immune components do not become fully functional until they reach four weeks of age or puberty (1). The innate immune responses, which are mediated by neutrophils and macrophages, do not fully develop until late gestation (2). The humoral elements present in calves, such as cytokines and complements, are significantly fewer compared to those found in adults (3). Peripheral blood T cells experience a significant decrease in numbers during the final month prior to birth, as they actively migrate and settle in the lymphoid tissues of fetal calves (4). The number of B cells are much lower in calves than adults (5). The number of dendritic cells is limited in neonates, and their antigen-presenting ability to activate the acquired immune system is delicate (6). The percentage of circulating natural killer cells increases from 3% to 10% of total lymphocytes between 1 to 8 weeks of age (4).

Calves are born with agammaglobulinemia and depend on the absorption of maternal immunoglobulins (Ig), primarily from colostrum, to ensure sufficient passive mucosal immunity after birth (7). The passive transfer of maternal Ig across the small intestine within the first 24 hours of birth serves to protect a calf from common pathogens until its own immature mucosal immune systems becomes fully functional (8). Importantly, the addition of sodium butyrate in milk tended to stimulate the concentration of circulating Ig in piglets (9). Passive immunity poses a dual challenge for unweaned calves. On one hand, it offers direct protection against diseases by providing immunoprotection. On the other hand, it can hinder the development of adaptive immunity establishment post vaccination (10). Multidrug-resistant *Escherichia coli* can also spread via colostrum feeding process among neonatal dairy calves (11). Acquiring immunoprotection through early vaccination is crucial for disease resistance in neonatal calves, especially considering the immense diversity and abundance of enteropathogenic microorganisms present in the environment. These microorganisms include bovine coronavirus, rotavirus, bovine viral diarrhea virus, *Salmonella enterica*, *Escherichia coli*, *Clostridium perfringens*, and Cryptosporidium parvum. Colostrum-fed preruminant calves are capable of generating robust adaptive mucosal and cellular immunity following early vaccination (12, 13).

The endogenous short-chain fatty acids (SCFA) are the primary metabolic end products of the fermentation of non-digestible carbohydrates (NDC), which include acetic acid, butyric acid, and propionic acid. They are widely distributed in colon. Considering that free forms of SCFA present a strong odor and their limiting uses in diet formulation, the beneficial exogeous types of SCFA mainly contained the infusions of both sodium acetate, sodium propionate, and sodium butyrate, which induced ameliorated antioxidant capacity, enhanced expression levels of occludin protein, and increased abundance of rumen bacteria, mainly including *Butyrivibrio*, *Rikenellaceae* RC9, and *Alloprevotella*. Sodium butyrate infusion can also strengthen antioxidant capacity, rumen and gut barrier functions (14). As for colonic digesta, the supplementation of butyrate precursors, such as gluconate, Ca-butyrate, have been shown to increase butyrate production in the GIT. Ca-butyrate increased *in vivo* ruminal acetate absorption and tended to increase *ex vivo* gut barrier function (15). The concentration of SCFA in the GIT, ranging from 20 to 140 mM, is primarily determined by several factors including the presence of microorganisms, transit time of intestinal substrate, metabolic flux of SCFA between the host and microbiota, and the fiber content of in the host diet (16). The production pathways of acetate are widely distributed among gut microbiota, whereas the production pathways of propionate and butyrate seem to be highly conserved and substrate specific in bacteria. Nowadays, metagenomic approaches facilitate characterization of bacteria accounting for SCFA production. Among these organisms, *Akkermansia municiphilla* has been identified as a key propionate producer (17). *Faecalibacterium prausnitzii*, *Eubacterium rectale*, *Eubacterium hallii* and *R. bromii* appear to be the primary organisms responsible for butyrate production (18). More studies are needed to focus on the relationships between dietary intake, gut microbiota diversity, and function, and their significances on calf intestinal health.

As important energy sources, SCFA salvage energy from NDC sources, which contribute 5% to 15% of the total caloric requirements (19). Apart from acting as local substrates for energy production, SCFA play a crucial role in maintaining intestinal mucosal immunity of human beings. They accomplish this by fortifying the barrier function of intestinal epithelial cells (IEC), primarily through enhancing the transcription of mucin genes in the goblet cells (20, 21). SCFA have been demonstrated their effectiveness in restraining the growth of pathogenic bacteria (22, 23). Furthermore, SCFA serve as important regulators of pro-inflammatory mucosal immune responses and the expansion of peripheral T cells (24–26).

The purpose of this review is to summarize recent findings on the functions of SCFA and the underlying mechanisms by which they ameliorate intestinal mucosal immunity in dairy calves. In addition, we aim to provide new insights into the effects of SCFA on immunoregulation and the interactions between gut microbiota, SCFA, and the intestinal mucosal immunity of dairy calves.

## The roles and mechanisms of SCFA in intestinal passive mucosal immunity

### Regulation of the gastrointestinal barrier function by SCFA

**Figure 1:**
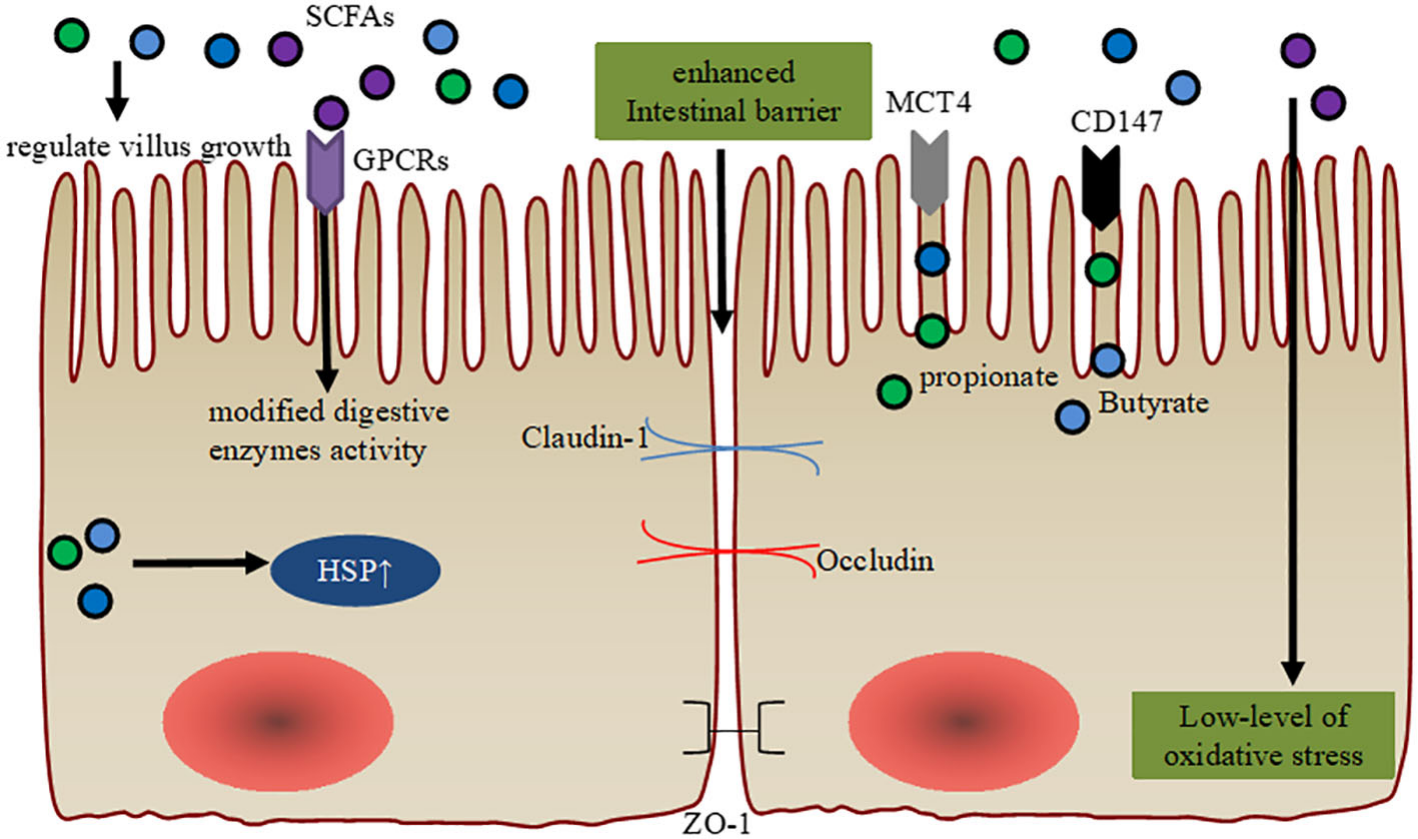
Maintenance of intestinal barrier function via SCFA. SCFA regulate intestinal villus growth and modified cellular digestive enzymes activity through GPCR. SCFA orchestrate the expression of the tight junction proteins (ZO-1, Occludin, and Claudin-1) to ameliorate intestinal barrier function. Intracellular SCFA stimulation of the cytoprotection through heat shock protein (HSP) and they have antioxidative stress function. Enhanced gene expression levels of MCT4 and CD147 are observed in the ruminal epithelial cells post-weaning, which are associated with the effects of diet-derived propionate and butyrate.

The maturation process of the gastrointestinal tract (GIT) has received significant attention in dairy calves as the first inherent barrier. In fact, the establishment of the innate gastrointestinal epithelial barrier is closely linked to SCFA, tryptophan and its derivatives, and some amino acids. (**Figure 1**) (27). Short-chain fatty acids, especially butyrate, play a significant role in the postnatal development of the ruminal epithelium in calves (28). A well functioning rumen epithelium could lead to improved calf performance at a younger age.

Previous studies have shown that butyrate supplementation can affect villus growth in pre-ruminant calves (29, 30). In fact, butyrate supplementation and intensive milk feeding stimulate body growth and affect GIT development in pre-weaning calves. Intensive butyrate supplementation induces an elevated growth of the small intestinal mucosa, but does not affect rumen development during the weaning period (31). Furthermore, extra additions of SCFA, especially acetate, propionate, and butyrate, improve digestibility and feed efficiency by enhancing GIT maturation, modified activity of digestive enzymes, and stimulating cytoprotection through the expression of heat shock protein (HSP) expression in young animals. Importantly, these results indicate an improved gut microbiota structure, including enriched *Butyricicoccus* and *Faecalibacterium* (27, 32, 33). Additionally, increased enterocyte proliferation in the upper jejunum and duodenal villi height were observed post sodium butyrate feeding in pre-weaning calves (29). Interestingly, administration of SCFA have also been found to increase the transcription of mucin genes in human intestinal epithelial goblet cells (20). SCFA or inulin supplementations, induce enriched α-defensin of Paneth cells *in vitro*, and that histone deacetylation (HDAC) and signal transducer and activator of transcription 3 (STAT3) might play a role in butyrate-mediated induction of α-defensins (34). Importantly, extra sodium butyrate addition provided more Na^+^ to the ruminal epithelium, which may help stabilize tissue integrity (35).

In other mammals, SCFA-producing microbes or SCFA induce goblet cell differentiation, mucus production, and high IEC integrity, thus maintaining colonic epithelial homeostasis (36, 37). Similarly, butyrate restoration can improve IEC junctional integrity in young mice (38). In weaning calves, sodium butyrate has been used to support expression of tight junction (claudin-1, claudin-4 or occludin) and tight junction-associated proteins (TJP, zonula occludens) in stratified squamous ruminal epithelium (39). Butyrate is also considered the most important regulator of TJP in human IEC cells, upregulating the expressions claudin-1 and zonula occludens-1 (ZO-1) (40).

Unprotected sodium butyrate supplementation (0.3% of dry matter or 45 g/d) has been proven to stimulate growth performance, feed efficiency, and GIT development in pre-weaned calves, and it can be recommended for practical use on dairy farms (30). Importantly, sodium butyrate supplementation increases glutathione peroxidase (GSH-Px) activity and decreases malondialdehyde (MDA) concentration among preweaning calves, helping them cope with the oxidative stress they experience in their young lives (41). Dietary SCFA supplementations in liquid or solid feed can promote GIT development in newborn calves (30). Furthermore, the increased gene expression levels of MCT4 and CD147 are observed in ruminal epithelial cells post-weaning, which are associated with the effects of propionate and butyrate derived from the diet (42). Thus, future studies should focus on comparing the effects of different sources and forms of SCFA on GIT development and calf performance to confirm their beneficial effects on the gastrointestinal barrier. On the other hand, low feed intake during heat stress, transportation, and infectious disease pose significant challenges to the ruminal epithelial barrier. Feed restriction increases the risk of post-restriction subacute ruminal acidosis in weaning calves, as it rapidly and dose-dependently decreases the absorption capacity for SCFA (39). Although oral butyrate is used in weaning calves to support ruminal barrier development, excessive butyrate intervention may promote hyperkeratosis, parakeratosis, and epithelial injury in the fully developed rumen of adult cows (39). Therefore, future research is urgently needed to enhance the understanding of appropriate SCFA concentrations and the maintenance of intestinal barrier function during the early life period of dairy calves.

### Facilitation of passive mucosal immunity progression and maintenance of the balance between intestinal immunity and diseases via SCFA

Since Ig are mostly obtained from colostrum, which acts as the only source of antibodies before calves begin to produce its own Ig in sufficient quantities. Proper management and improvement of passive transfer of Ig from dam to newborn calves have been reported to play a vital role in determining the health of these young animals (43). It is known that passive transfer of maternal IgG is facilitated by receptor-mediated endocytosis via the epithelial FcRn receptor and endocytosis using “transport vacuoles” (44, 45). However, as calves mature, epithelial cell endocytosis slowly diminishes. Maternal macromolecule passage over the GIT is largely suppressed in neonatal animals as they grow (46). Currently, the threshold for passive transfer of immunity is a blood IgG concentration of 10 mg/mL or a serum total protein concentration of 5.2 to 5.5 g/dL in the first 2 days of neonatal calves (47). The promotion of passive transfer of innate immunity and the prevention of failure in passive immunity transfer are the underlying reasons for feeding dairy colostrum. In fact, dietary butyrate supplementation has been shown to improve the IgG concentration in porcine colostrum. Butyrate addition in the milk also tends to stimulate the circulating IgA concentration in piglets. This is attributed to the fact that the serum IgG concentration and IgA-positive plasma cell count in the jejunum from pigs fed sodium butyrate were significantly higher than those given the basal diet (9, 48, 49). Importantly, SCFA promote B-cell IgA class switching and intestinal IgA production via the GPR43 of dendritic cells (DCs) in mice (50). However, direct butyrate supplementation, including rumen-protected butyrate and calcium-sodium-butyrate, did not affect serum immunoglobulin concentrations in pre-weaning calves (43, 51). While, feed supplementation with mulberry leaf flavonoids increased the total volatile fatty acid and propionate concentrations in pre-weaning and post-weaning calves, thus inducing enhanced serum concentrations of IgG and IgA (52). Supplementing lambs with Rosmarinus officinalis leaves or Chinese medicine polysaccharides had greater serum IgG and IgA compared to control groups (53, 54). This discrepancies may be attributable to differences in animal species and the assumed fact that SCFA had no direct effect on B-cell IgA or IgG class switching and intestinal IgA or IgG production. Butyrate may reduce IgG absorption by increasing the rate of cell differentiation, thus inducing early maturation of epithelial cells in newborn calves (43). Previous studies also demonstrate that maternal antibody transfer has extra-immunological effects in addition to the classical protective immune effects. These extra effects mainly include direct effects on intestinal growth and other organs in neonatal animals, especially on GIT structure, enteric nervous system, hippocampal development and behavior of animals (55, 56).

In fact, SCFA and IgA supplement each other. The secretory IgA (sIgA) isotype is the most abundant Ig in mucosal secretions and accounts for about 7% of total Ig composition in dairy colostrum (57). Secretory IgA favors the development of commensal bacteria in mice, especially the enriched SCFA-producing bacteria in the gut lumen, and also provides sufficient protection against enteric pathogens through its mucus-binding properties (58, 59). Additionally, the colonization of SCFA-producing bacteria is beneficial in defending against pathogens, stimulating commensal bacteria colonization on the mucosal surface and inducing enriched SCFA in the gut lumen. SCFA-producing bacteria, especially acetate-producing gut bacteria, induced IgA production mainly by the activation of GPR43 (G protein-coupled receptor 43) and cytosolic cGAS-STING pathway (60). While, endogenous IgA cannot reach a functional concentration (1 mg/mL) before 8 days of calf age, and appreciable blood concentration of IgA is only detected 16 days after birth (10, 61). Many schemes have been used to stimulate IgA production. Interestingly, direct Saccharomyces cerevisiae boulardii (SCB) supplementation or the mutual interaction between SCB and bacteria is responsible for IgA production and early bacterial colonization in the GIT of neonatal calves (62). This is attributed to gut microbiota improvement, with reduced *E. coli* and enriched *Fecalibacterium* in the hindgut, thus inducing higher production of SCFA in SCB treatment groups.

As an important part of innate immunity, antimicrobial peptides (AMP) secreted by IEC play a crucial role in regulating intestinal homeostasis by controlling intestinal microflora populations. Butyrate can boost AMP production, such as defensin and regenerative islet derived protein III γ in the IECs of mice through the SCFA receptor GPR43. Furthermore, butyrate also improves the expression of porcine β-defensin-2 and β-defensin-3 (27).

The host’s innate responses to pathogens hold the balance between intestinal immunity and diseases. SCFA show promise in the prevention and treatment of intestinal diseases in human health applications and have multi-faceted roles in different metabolic systems (63). It has been demonstrated that SCFA supplementation enhanced IL-4 and immunoglobulin productions in response to challenges, accompanied by enhanced titers for bovine viral diarrhea and respiratory parainfluenza-3. Additionally, supplementation of milk replacer with a blend of butyric acid increases antibody responses and improves growth and feed efficiency in pre-weaning calves. Moreover, alterations in IL-4 mRNA expression levels are closely related to the humoral immune responses of calves (64), highlighting the feasibility of SCFA as novel immunoregulators. This assertion is based on the fact that IL-4 promote the differentiation of T and B cells in Ig synthesis. IL-4 induces IgE and IgG4 secretion by B cells in peripheral blood mononuclear cells (PBMC) of humans (65). Butyrate administration suppresses nuclear factor kappaB (NF-κB) activation in macrophages and also induces the inhibition of histone deacetylase (HDAC) in acute myeloid leukemia in humans (66, 67).

However, to date, the exact mechanisms underlying these immune effects are still unclear. Elucidating the immunomodulatory mechanisms of SCFA in dairy calves will help unravel the advantages of SCFA supplementation and provide clues for preventing and controlling diarrhea and pneumonia.

## Maintenance of gut microbiota homeostasis by SCFA

During the first few weeks or months of a calf’s life, there is a significant change in their digestive physiology as they transition from being a simple monogastric animal to a fully functional ruminant (68). Unfortunately, calf intestinal diseases have a major impact on productivity and result in substantial economic losses for dairy operations. Out of these diseases, calf diarrhea and other digestive problems are the primary contributors to pre-weaned calf mortality (69, 70).

Proper colonization of microbiota plays a crucial role in the development of the immune system and the establishment of the GIT structure. It also helps neonatal calves develop resistance against pathogenic challenges and creates a functional fermentation environment (71). Additionally, more than 20% of milk solids reach the hindgut during the milk feeding phase, emphasizing the importance of hindgut microbiota in dairy calves (72). The hindgut microbiota’s significance on feed fermentation during the pre-weaning period is indicated by the upregulation of predicted microbial genes involved in energy metabolism, amino acids metabolism, and carbohydrate metabolism (68). The close alignment between SCFA, mucosa-attached carbohydrate utilizing microbiota (such as *Coprococcus* 1, *Blautia*, and *Lachnospiraceae* NC2004 group), and pathogenic bacteria (*Escherichia-Shigella* and *Salmonella*) further highlights the importance of hindgut microbiota in fermentation process during the pre-weaning period (73). The presence of *Butyricicoccus*, *Faecalibacterium*, *Collinsella*, and *Coriobacterium*, key commensal bacteria of healthy newborn calves, is positively related to high production of unabsorbed carbohydrates, SCFA, and other prebiotics (74). Tributyrin supplementation significantly increased the abundance of short-chain fatty acid (SCFA)-producing bacteria, including *Ruminococcaceae*, *Lachnospiraceae*, *Prevotella* and *Rikenellaceae.* This increase was negatively associated with TLR2 and IL-1β expressions, but positively linked to intestinal barrier genes expressions (75). Besides, the molar proportion of SCFA have the positive correlation with colon mucosa-associated beneficial bacteria, indicating that SCFA might play an important role in maintaining the gut health of 2-d-old calves (76). Sodium butyrate has also shown the instructive effects on growth and performance occur in tandem with changes in the abundance of health-associated bacteria in the hindgut of milk-fed calves (77).

During the first month of life, milk-fed preruminant calves have a similar number of colonized bacterial species in the rumen and colon. The variation of colonic bacterial composition significantly diminishes by four weeks of age (78). Lactic acid bacteria, such as *Lactobacillus*, *Streptococcus*, *Enterococcus*, and *Bifidobacterium*, dominate the microbial community in the hindgut. High SCFA concentrations may inhibit the abundance of genus *Bacteroides*, which could be beneficial for intestinal health and survival of the neonatal calves in the early weeks of life (72).

As an important metabolite of gut microbiota, SCFA have great potential as feed additives to ameliorate the gut microbiota species and community of calves. However, there are still many controversies regarding their effects on the early colonizations of microorganisms.

## Ameliorations of intestinal inflammatory reaction and protective immunity via SCFA

**Figure 2:**
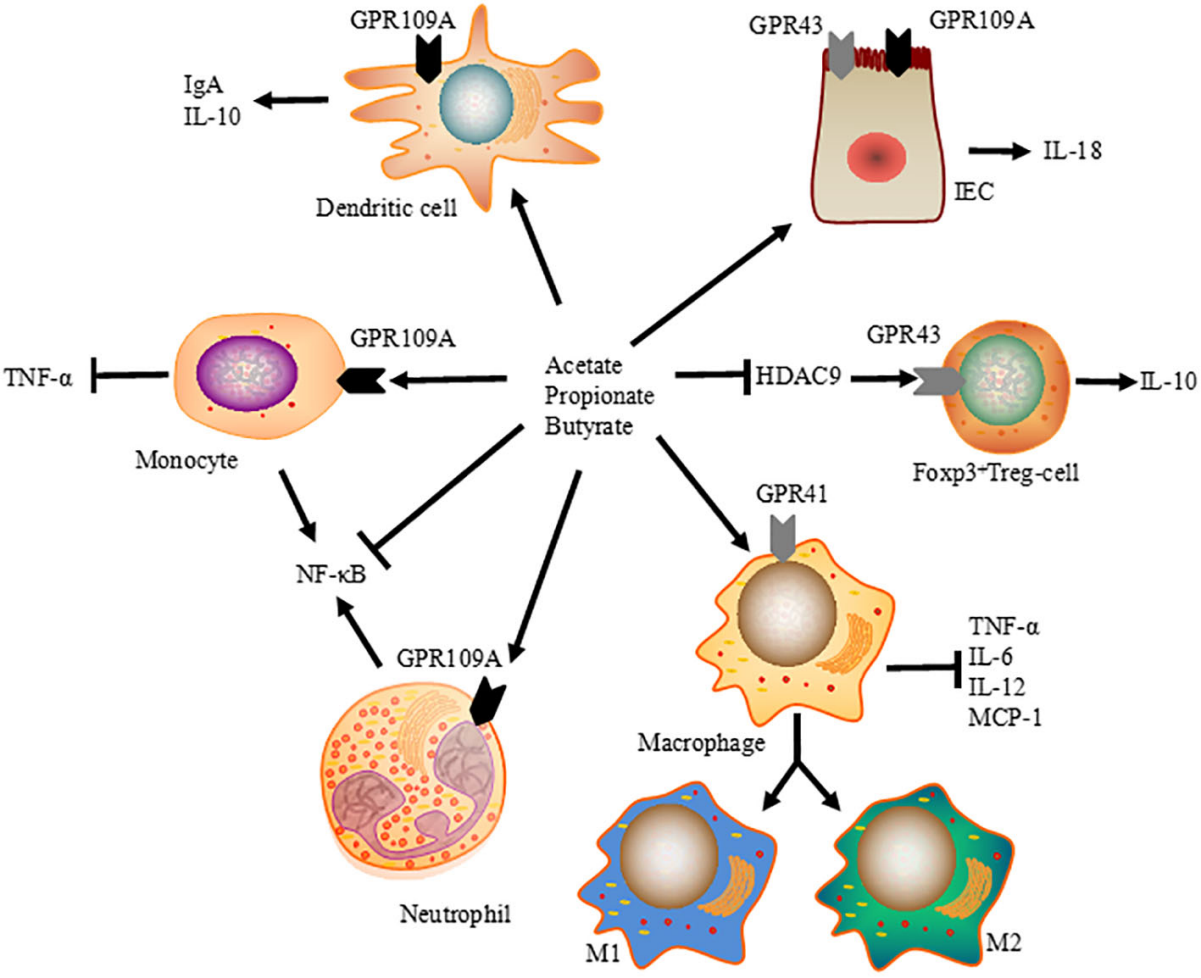
Immunomodulatory function of SCFA on intestinal immune system. SCFA modulate tissue inflammation and protective immunity by promoting the differentiation of Foxp3 positive Treg cells, promoting intestinal IgA and IL-10 expressions via dendritic cells, and enhancing IL-18 production in IEC. At the same time, SCFA-induced HDAC inhibition is a crucial regulator of NF-κB activity, reducing the expression of TNF-α, IL-6, IL-12, and MCP-1 in macrophages, and ameliorating TNF-α production in monocytes.

Previous studies have revealed the regulatory function of SCFA in intestinal immune system (**Figure 2**). In most cases, they act as signaling molecules that promote tolerogenic and anti-inflammatory cell responses by inhibiting HDAC, which results in inactivated nuclear factor-kappaB (NF-κB) and downregulation of tumor necrosis factor (TNF) production in mammals (24, 25). The inhibition of HDAC by SCFA is a crucial regulator of NF-κB activity and pro-inflammatory immune responses. A cohort study found a higher prevalence of SCFA-producing bacteria belonging to *Ruminococcaceae* and *Lachnospiraceae* in healthy neonatal calves, with an enriched presence of butyric acid compared to the bacterial enteritis group (79). Additionally, in mice, the binding of SCFA to GPR43 and GPR109A in IEC activates inflammasome assembly and enhances the downstream inflammatory cytokine IL-18 (80).

SCFA contain volatile species with short half-lives and rapid metabolism, and only adequate amounts of SCFA are sufficient to trigger HDAC activation in human colonic cells (81). However, their effects may require specific transporters since SCFA can also suppress HDAC through GPCR-dependent mechanisms in mammals (82, 83). Therefore, further studies are needed to investigate the immunoregulatory functions and therapeutic potentials of SCFA in dairy calves.

Previous reports have show that SCFA influence the proliferation of peripheral T cells, especially regulatory T cells (Treg), in the mucosa lamina propria through HDAC inhibition. In mice, inhibition of HDAC9 upregulates the expression of forkhead box P3 (FOXP3) and expands FOXP3 ^+^Treg cells (84). Therefore, giving mice a high-fiber or SCFA-supplemented diet not only eliminates colonic inflammation but also suppresses allergic airway diseases by increasing the suppressive activity of FOXP3 ^+^Treg cells (85). Orally administered SCFA induced the activation of both effector (Th1 and Th17) and regulatory T cells in ureter and kidney tissues of young mice, leading to T cell-mediated ureteritis and kidney hydronephrosis. Furthermore, systemically administration of SCFA at higher than physiological levels can cause dysregulated T cell responses and tissue inflammation in the renal system of mice (86). Thus, in addition to their immunological effects, the pathological effects of chronically elevated SCFA should also be taken seriously.

Considering that SCFA are ligands for GPCR, many studies have also explored other mechanisms of SCFA-induced GPCR engagement. GPCR is expressed by numerous cell types, including IEC, dendritic cells, and T cells (87). GPR43 expression has been found to be critical for the expansion and suppressive function of Treg cells (26). Additionally, both niacin and butyrate acid can prevent colitis and colon carcinogenesis by upregulating anti-inflammatory molecules secreted by monocytes, promoting differentiation of Treg cells and interleukin-10 (IL-10)-producing T cells (88). Gallic acid has been shown to mediated colitis attenuation through the upregulation of hindgut acetate and butyrate, with elevated expression of IL-10 and TGF-β in newborn calves (89). Therefore, the immunoregulatory effects of SCFA largely depend on the context and cell types, allowing the host to monitor pro-inflammatory immune responses and maintain mucosal immune homeostasis.

## Conclusions and future perspectives

Previous publications have revealed the beneficial effects of SCFA on GIT maturation, the transfer of passive mucosal immunity, microbiota homeostasis, and the moderation of immune responses. SCFA, which provide energy for microbes and strengthen the expansion of IEC, have been found to establish a mutual relationship with the host. in dairy calves. This interaction accelerates the fermentation of undigested complex carbohydrates, leading to the maintenance of microbial communities compositions and homeostasis of host’s mucosal immunity.

In fact, SCFA and their derivatives show promise in treating human diseases, particularly inflammatory bowel diseases. Therefore, the application of SCFA as feed additives in calf nutrition is very promising, as they hold potential as replacements for certain antibiotic growth promoters. Further research on SCFA may help in developing valuable supplements and providing alternatives to antibiotics in the dairy industry.

## Author contributions

ZH: Funding acquisition, Writing – original draft, Writing – review & editing. HD: Supervision, Writing – review & editing.

## References

[1] Reber AJ Lockwood A Hippen AR Hurley DJ. Colostrum induced phenotypic and trafficking changes in maternal mononuclear cells in a peripheral blood leukocyte model for study of leukocyte transfer to the neonatal calf. Vet Immunol Immunopathol (2006) 109:139–50. doi: 10.1016/j.vetimm.2005.08.014 16169602

[2] Barrington GM Parish SM. Bovine neonatal immunology. Vet Clin North Am Food Anim Pract (2001) 17:463–76. doi: 10.1016/s0749-0720(15)30001-3 PMC7135619 11692503

[3] Charleston B Fray MD Baigent S Carr BV Morrison WI. Establishment of persistent infection with non-cytopathic bovine viral diarrhea virus in cattle is associated with a failure to induce type I interferon. J Gen Virol (2001) 82:1893–7. doi: 10.1099/0022-1317-82-8-1893 11457995

[4] Kampen AH Olsen I Tollersrud T Storset AK Lund A. Lymphocyte subpopulations and neutrophil function in calves during the first 6 months of life. Vet Immunol Immunopathol (2006) 113:53–63. doi: 10.1016/j.vetimm.2006.04.001 16772096

[5] Senogles DR Paul PS Johnson DW Muscoplat CC. Ontogeny of T cells, B cells and monocytes in the bovine fetus. Clin Exp Immunol (1979) 36:299–303. 314369 PMC1537733

[6] Morein B Abusugra I Blomqvist G. Immunity in neonates. Vet Immunol Immunopathol (2002) 87:207–13. doi: 10.1016/s0165-2427(02)00078-8 12072236

[7] McGuirk SM Collins M. Managing the production, storage, and delivery of colostrum. Vet Clin North Am Food Anim Pract (2004) 20:593–603. doi: 10.1016/j.cvfa.2004.06.005 15471626

[8] Godden SM Lombard JE Woolums AR. Colostrum management for dairy calves. Vet Clin North Am Food Anim Pract (2019) 35:535–56. doi: 10.1016/j.cvfa.2019.07.005 PMC7125574 31590901

[9] Fang CL Sun H Wu J Niu HH Feng J. Effects of sodium butyrate on growth performance, hematological and immunological characteristics of weanling piglets. J Anim Physiol Anim Nutr (Berl) (2014) 98:680–5. doi: 10.1111/jpn.12122 24024579

[10] Chase CC Hurley DJ Reber AJ. Neonatal immune development in the calf and its impact on vaccine response. Vet Clin North Am Food Anim Pract (2008) 24:87–104. doi: 10.1016/j.cvfa.2007.11.001 18299033 PMC7127081

[11] He Z Yang S Ma Y Zhang S Cao Z. Detection of CTX-M-15 Extended-Spectrum beta-Lactamases Producing Escherichia coli Isolates from Colostrum and Faces of Newborn Dairy Calves in China. Pathogens (2021) 10. doi: 10.3390/pathogens10091162 PMC8466790 34578194

[12] Nonnecke BJ Waters WR Goff JP Foote MR. Adaptive immunity in the colostrum-deprived calf: Response to early vaccination with Mycobacterium bovis strain bacille Calmette Guerin and ovalbumin. J Dairy Sci (2012) 95:221–39. doi: 10.3168/jds.2011-4712 22192201

[13] Cho YI Yoon KJ. An overview of calf diarrhea - infectious etiology, diagnosis, and intervention. J Vet Sci (2014) 15:1–17. doi: 10.4142/jvs.2014.15.1.1 24378583 PMC3973752

[14] Zhen Y Xi Z Nasr SM He F Han M Yin J. Multi-omics reveals the impact of exogenous short-chain fatty acid infusion on rumen homeostasis: insights into crosstalk between the microbiome and the epithelium in a goat model. Microbiol Spectr (2023) 11:e534322. doi: 10.1128/spectrum.05343-22 PMC10433986 37439665

[15] Watanabe D Doelman J Steele MA Guan LL Seymour DJ Penner GB. A comparison of post-ruminal provision of Ca-gluconate and Ca-butyrate on growth performance, gastrointestinal barrier function, short-chain fatty acid absorption, intestinal histology, and brush-border enzyme activity in beef heifers. J Anim Sci (2023) 101. doi: 10.1093/jas/skad050 PMC10022388 36799118

[16] Cummings JH Pomare EW Branch WJ Naylor CP Macfarlane GT. Short chain fatty acids in human large intestine, portal, hepatic and venous blood. Gut (1987) 28:1221–7. doi: 10.1136/gut.28.10.1221 PMC1433442 3678950

[17] Derrien M Vaughan EE Plugge CM de Vos WM. Akkermansia muciniphila gen. Nov., Sp. Nov., A human intestinal mucin-degrading bacterium. Int J Syst Evol Microbiol (2004) 54:1469–76. doi: 10.1099/ijs.0.02873-0 15388697

[18] Louis P Young P Holtrop G Flint HJ. Diversity of human colonic butyrate-producing bacteria revealed by analysis of the butyryl-CoA:acetate CoA-transferase gene. Environ Microbiol (2010) 12:304–14. doi: 10.1111/j.1462-2920.2009.02066.x 19807780

[19] Bergman EN. Energy contributions of volatile fatty acids from the gastrointestinal tract in various species. Physiol Rev (1990) 70:567–90. doi: 10.1152/physrev.1990.70.2.567 2181501

[20] Willemsen LE Koetsier MA van Deventer SJ van Tol EA. Short chain fatty acids stimulate epithelial mucin 2 expression through differential effects on prostaglandin E(1) and E(2) production by intestinal myofibroblasts. Gut (2003) 52:1442–7. doi: 10.1136/gut.52.10.1442 PMC1773837 12970137

[21] Gaudier E Jarry A Blottiere HM de Coppet P Buisine MP Aubert JP. Butyrate specifically modulates MUC gene expression in intestinal epithelial goblet cells deprived of glucose. Am J Physiol Gastrointest Liver Physiol (2004) 287:G1168–74. doi: 10.1152/ajpgi.00219.2004 15308471

[22] Kabara JJ Swieczkowski DM Conley AJ Truant JP. Fatty acids and derivatives as antimicrobial agents. Antimicrob Agents Chemother (1972) 2:23–8. doi: 10.1128/aac.2.1.23 PMC444260 4670656

[23] Thormar H Hilmarsson H Bergsson G. Stable concentrated emulsions of the 1-monoglyceride of capric acid (monocaprin) with microbicidal activities against the food-borne bacteria Campylobacter jejuni, Salmonella spp., And Escherichia coli. Appl Environ Microbiol (2006) 72:522–6. doi: 10.1128/AEM.72.1.522-526.2006 PMC1352223 16391087

[24] Vinolo MA Rodrigues HG Hatanaka E Sato FT Sampaio SC Curi R. Suppressive effect of short-chain fatty acids on production of proinflammatory mediators by neutrophils. J Nutr Biochem (2011) 22:849–55. doi: 10.1016/j.jnutbio.2010.07.009 21167700

[25] Usami M Kishimoto K Ohata A Miyoshi M Aoyama M Fueda Y. Butyrate and trichostatin a attenuate nuclear factor kappaB activation and tumor necrosis factor alpha secretion and increase prostaglandin E2 secretion in human peripheral blood mononuclear cells. Nutr Res (2008) 28:321–8. doi: 10.1016/j.nutres.2008.02.012 19083427

[26] Smith PM Howitt MR Panikov N Michaud M Gallini CA Bohlooly-Y M. The microbial metabolites, short-chain fatty acids, regulate colonic Treg cell homeostasis. Science (2013) 341:569–73. doi: 10.1126/science.1241165 PMC3807819 23828891

[27] Wu L Tang Z Chen H Ren Z Ding Q Liang K. Mutual interaction between gut microbiota and protein/amino acid metabolism for host mucosal immunity and health. Anim Nutr (2021) 7:11–6. doi: 10.1016/j.aninu.2020.11.003 PMC8110859 33997326

[28] Sakata T Tamate H. Rumen epithelial cell proliferation accelerated by rapid increase in intraruminal butyrate. J Dairy Sci (1978) 61:1109–13. doi: 10.3168/jds.S0022-0302(78)83694-7 721981

[29] Guilloteau P Zabielski R David JC Blum JW Morisset JA Biernat M. Sodium-butyrate as a growth promoter in milk replacer formula for young calves. J Dairy Sci (2009) 92:1038–49. doi: 10.3168/jds.2008-1213 19233797

[30] Gorka P Kowalski ZM Zabielski R Guilloteau P. Invited review: Use of butyrate to promote gastrointestinal tract development in calves. J Dairy Sci (2018) 101:4785–800. doi: 10.3168/jds.2017-14086 29525310

[31] Koch C Gerbert C Frieten D Dusel G Eder K Zitnan R. Effects of ad libitum milk replacer feeding and butyrate supplementation on the epithelial growth and development of the gastrointestinal tract in Holstein calves. J Dairy Sci (2019) 102:8513–26. doi: 10.3168/jds.2019-16328 31255268

[32] Lingbeek MM Borewicz K Febery E Han Y Doelman J van Kuijk S. Short-chain fatty acid administration via water acidifier improves feed efficiency and modulates fecal microbiota in weaned piglets. J Anim Sci (2021) 99. doi: 10.1093/jas/skab307 PMC8599185 34679178

[33] Yusuf F Adewiah S Fatchiyah F. The level short chain fatty acids and HSP 70 in colorectal cancer and Non-Colorectal cancer. Acta Inform Med (2018) 26:160–3. doi: 10.5455/aim.2018.26.160-163 PMC6195403 30515005

[34] Beisner J Filipe RL Kaden-Volynets V Stolzer I Gunther C Bischoff SC. Prebiotic inulin and sodium butyrate attenuate Obesity-Induced intestinal barrier dysfunction by induction of antimicrobial peptides. Front Immunol (2021) 12:678360. doi: 10.3389/fimmu.2021.678360 34177920 PMC8226265

[35] Bertens CA Mutsvangwa T Van Kessel AG Penner GB. Effect of sodium concentration and mucosal pH on apical uptake of acetate and butyrate, and barrier function of the isolated bovine ruminal epithelium. J Dairy Sci (2023) 106:7310–9. doi: 10.3168/jds.2022-23052 37210365

[36] Wrzosek L Miquel S Noordine ML Bouet S Joncquel CM Robert V. Bacteroides thetaiotaomicron and Faecalibacterium prausnitzii influence the production of mucus glycans and the development of goblet cells in the colonic epithelium of a gnotobiotic model rodent. BMC Biol (2013) 11:61. doi: 10.1186/1741-7007-11-61 23692866 PMC3673873

[37] Fukuda S Toh H Hase K Oshima K Nakanishi Y Yoshimura K. Bifidobacteria can protect from enteropathogenic infection through production of acetate. Nature (2011) 469:543–7. doi: 10.1038/nature09646 21270894

[38] Mathewson ND Jenq R Mathew AV Koenigsknecht M Hanash A Toubai T. Gut microbiome-derived metabolites modulate intestinal epithelial cell damage and mitigate graft-versus-host disease. Nat Immunol (2016) 17:505–13. doi: 10.1038/ni.3400 PMC4836986 26998764

[39] Aschenbach JR Zebeli Q Patra AK Greco G Amasheh S Penner GB. Symposium review: The importance of the ruminal epithelial barrier for a healthy and productive cow. J Dairy Sci (2019) 102:1866–82. doi: 10.3168/jds.2018-15243 30580938

[40] Wang HB Wang PY Wang X Wan YL Liu YC. Butyrate enhances intestinal epithelial barrier function via up-regulation of tight junction protein Claudin-1 transcription. Dig Dis Sci (2012) 57:3126–35. doi: 10.1007/s10620-012-2259-4 22684624

[41] Liu W La ATZ Evans A Gao S Yu Z Bu D. Supplementation with sodium butyrate improves growth and antioxidant function in dairy calves before weaning. J Anim Sci Biotechnol (2021) 12:2. doi: 10.1186/s40104-020-00521-7 33397482 PMC7780688

[42] Nakamura S Haga S Kimura K Matsuyama S. Propionate and butyrate induce gene expression of monocarboxylate transporter 4 and cluster of differentiation 147 in cultured rumen epithelial cells derived from preweaning dairy calves. J Anim Sci (2018) 96:4902–11. doi: 10.1093/jas/sky334 PMC6247864 30215729

[43] Hiltz RL Laarman AH. Effect of butyrate on passive transfer of immunity in dairy calves. J Dairy Sci (2019) 102:4190–7. doi: 10.3168/jds.2018-15555 30879822

[44] Israel EJ Patel VK Taylor SF Marshak-Rothstein A Simister NE. Requirement for a beta 2-microglobulin-associated Fc receptor for acquisition of maternal IgG by fetal and neonatal mice. J Immunol (1995) 154:6246–51. doi: 10.4049/jimmunol.154.12.6246 7759862

[45] Baintner K. Transmission of antibodies from mother to young: Evolutionary strategies in a proteolytic environment. Vet Immunol Immunopathol (2007) 117:153–61. doi: 10.1016/j.vetimm.2007.03.001 17459489

[46] Roopenian DC Akilesh S. FcRn: The neonatal Fc receptor comes of age. Nat Rev Immunol (2007) 7:715–25. doi: 10.1038/nri2155 17703228

[47] Windeyer MC Leslie KE Godden SM Hodgins DC Lissemore KD LeBlanc SJ. Factors associated with morbidity, mortality, and growth of dairy heifer calves up to 3 months of age. Prev Vet Med (2014) 113:231–40. doi: 10.1016/j.prevetmed.2013.10.019 24269039

[48] He B Wang M Guo H Jia Y Yang X Zhao R. Effects of sodium butyrate supplementation on reproductive performance and colostrum composition in gilts. Animal (2016) 10:1722–7. doi: 10.1017/S1751731116000537 27040131

[49] Chen J Xu Q Li Y Tang Z Sun W Zhang X. Comparative effects of dietary supplementations with sodium butyrate, medium-chain fatty acids, and n-3 polyunsaturated fatty acids in late pregnancy and lactation on the reproductive performance of sows and growth performance of suckling piglets. J Anim Sci (2019) 97:4256–67. doi: 10.1093/jas/skz284 PMC6776281 31504586

[50] Wu W Sun M Chen F Cao AT Liu H Zhao Y. Microbiota metabolite short-chain fatty acid acetate promotes intestinal IgA response to microbiota which is mediated by GPR43. Mucosal Immunol (2017) 10:946–56. doi: 10.1038/mi.2016.114 PMC5471141 27966553

[51] Gerbert C Frieten D Koch C Dusel G Eder K Stefaniak T. Effects of ad libitum milk replacer feeding and butyrate supplementation on behavior, immune status, and health of Holstein calves in the postnatal period. J Dairy Sci (2018) 101:7348–60. doi: 10.3168/jds.2018-14542 29778472

[52] Kong L Yang C Dong L Diao Q Si B Ma J. Rumen fermentation characteristics in pre- and Post-Weaning calves upon feeding with mulberry leaf flavonoids and candida tropicalis individually or in combination as a supplement. Anim (Basel) (2019) 9:990. doi: 10.3390/ani9110990 PMC6912756 31752155

[53] Chen H Guo B Yang M Luo J Hu Y Qu M. Response of growth performance, blood biochemistry indices, and rumen bacterial diversity in lambs to diets containing supplemental probiotics and chinese medicine polysaccharides. Front Vet Sci (2021) 8:681389. doi: 10.3389/fvets.2021.681389 34250066 PMC8264418

[54] Odhaib KJ Adeyemi KD Ahmed MA Jahromi MF Jusoh S Samsudin AA. Influence of Nigella sativa seeds, Rosmarinus officinalis leaves and their combination on growth performance, immune response and rumen metabolism in Dorper lambs. Trop Anim Health Prod (2018) 50:1011–23. doi: 10.1007/s11250-018-1525-7 29654500

[55] Westrom B Arevalo SE Pierzynowska K Pierzynowski SG Perez-Cano FJ. The immature gut barrier and its importance in establishing immunity in newborn mammals. Front Immunol (2020) 11:1153. doi: 10.3389/fimmu.2020.01153 32582216 PMC7296122

[56] Wolinski J Slupecka M Westrom B Prykhodko O Ochniewicz P Arciszewski M. Effect of feeding colostrum versus exogenous immunoglobulin G on gastrointestinal structure and enteric nervous system in newborn pigs. J Anim Sci (2012) 90 Suppl 4:327–30. doi: 10.2527/jas.53926 23365369

[57] Stelwagen K Carpenter E Haigh B Hodgkinson A Wheeler TT. Immune components of bovine colostrum and milk. J Anim Sci (2009) 87:3–9. doi: 10.2527/jas.2008-1377 18952725

[58] Johansen FE Pekna M Norderhaug IN Haneberg B Hietala MA Krajci P. Absence of epithelial immunoglobulin a transport, with increased mucosal leakiness, in polymeric immunoglobulin receptor/secretory component-deficient mice. J Exp Med (1999) 190:915–22. doi: 10.1084/jem.190.7.915 PMC2195652 10510081

[59] Strugnell RA Wijburg OL. The role of secretory antibodies in infection immunity. Nat Rev Microbiol (2010) 8:656–67. doi: 10.1038/nrmicro2384 20694027

[60] Yu T Yang W Yao S Yu Y Wakamiya M Golovko G. STING promotes intestinal IgA production by regulating acetate-producing bacteria to maintain host-microbiota mutualism. Inflammation Bowel Dis (2023) 29:946–59. doi: 10.1093/ibd/izac268 PMC10233729 36661414

[61] Husband AJ Lascelles AK. Antibody responses to neonatal immunization in calves. Res Vet Sci (1975) 18:201–7. doi: 10.1016/S0034-5288(18)33614-2 805467

[62] Villot C Chen Y Pedgerachny K Chaucheyras-Durand F Chevaux E Skidmore A. Early supplementation of Saccharomyces cerevisiae boulardii CNCM I-1079 in newborn dairy calves increases IgA production in the intestine at 1 week of age. J Dairy Sci (2020) 103:8615–28. doi: 10.3168/jds.2020-18274 32684462

[63] Morrison DJ Preston T. Formation of short chain fatty acids by the gut microbiota and their impact on human metabolism. Gut Microbes (2016) 7:189–200. doi: 10.1080/19490976.2015.1134082 26963409 PMC4939913

[64] Hill TM Vandehaar MJ Sordillo LM Catherman DR Bateman HN Schlotterbeck RL. Fatty acid intake alters growth and immunity in milk-fed calves. J Dairy Sci (2011) 94:3936–48. doi: 10.3168/jds.2010-3935 21787930

[65] Ninomiya C Spiegelberg HL. IL-4 and transforming growth factor-beta suppress human immunoglobulin secretion in *vitro* by surface IgD- B cells. Clin Exp Immunol (1992) 89:261–8. doi: 10.1111/j.1365-2249.1992.tb06942.x PMC1554426 1638770

[66] Luhrs H Gerke T Muller JG Melcher R Schauber J Boxberge F. Butyrate inhibits NF-kappaB activation in lamina propria macrophages of patients with ulcerative colitis. Scand J Gastroenterol (2002) 37:458–66. doi: 10.1080/003655202317316105 11989838

[67] Maeda T Towatari M Kosugi H Saito H. Up-regulation of costimulatory/adhesion molecules by histone deacetylase inhibitors in acute myeloid leukemia cells. Blood (2000) 96:3847–56. doi: 10.1182/blood.V96.12.3847 11090069

[68] Steele MA Penner GB Chaucheyras-Durand F Guan LL. Development and physiology of the rumen and the lower gut: Targets for improving gut health. J Dairy Sci (2016) 99:4955–66. doi: 10.3168/jds.2015-10351 26971143

[69] Barrington GM Gay JM Evermann JF. Biosecurity for neonatal gastrointestinal diseases. Vet Clin North Am Food Anim Pract (2002) 18:7–34. doi: 10.1016/s0749-0720(02)00005-1 12064170 PMC7135474

[70] Barkley JA Pempek JA Bowman AS Nolting JM Lee J Lee S. Longitudinal health outcomes for enteric pathogens in preweaned calves on Ohio dairy farms. Prev Vet Med (2021) 190:105323. doi: 10.1016/j.prevetmed.2021.105323 33756433

[71] Yeoman CJ White BA. Gastrointestinal tract microbiota and probiotics in production animals. Annu Rev Anim Biosci (2014) 2:469–86. doi: 10.1146/annurev-animal-022513-114149 25384152

[72] Castro JJ Gomez A White B Loften JR Drackley JK. Changes in the intestinal bacterial community, short-chain fatty acid profile, and intestinal development of preweaned Holstein calves. 2. Effects of gastrointestinal site and age. J Dairy Sci (2016) 99:9703–15. doi: 10.3168/jds.2016-11007 27720148

[73] Song Y Malmuthuge N Steele MA Guan LL. Shift of hindgut microbiota and microbial short chain fatty acids profiles in dairy calves from birth to pre-weaning. FEMS Microbiol Ecol (2018) 94. doi: 10.1093/femsec/fix179 29267960

[74] He Z Ma Y Yang S Zhang S Liu S Xiao J. Gut microbiota-derived ursodeoxycholic acid from neonatal dairy calves improves intestinal homeostasis and colitis to attenuate extended-spectrum beta-lactamase-producing enteroaggregative Escherichia coli infection. Microbiome (2022) 10:79. doi: 10.1186/s40168-022-01269-0 35643532 PMC9142728

[75] Liu S Wu J Wu Z Alugongo GM Zahoor KM Li J. Tributyrin administration improves intestinal development and health in pre-weaned dairy calves fed milk replacer. Anim Nutr (2022) 10:399–411. doi: 10.1016/j.aninu.2022.06.004 35949196 PMC9356024

[76] Ma T O’Hara E Song Y Fischer AJ He Z Steele MA. Altered mucosa-associated microbiota in the ileum and colon of neonatal calves in response to delayed first colostrum feeding. J Dairy Sci (2019) 102:7073–86. doi: 10.3168/jds.2018-16130 31202657

[77] O’Hara E Kelly A McCabe MS Kenny DA Guan LL Waters SM. Effect of a butyrate-fortified milk replacer on gastrointestinal microbiota and products of fermentation in artificially reared dairy calves at weaning. Sci Rep (2018) 8:14901. doi: 10.1038/s41598-018-33122-6 30297834 PMC6175921

[78] Meale SJ Chaucheyras-Durand F Berends H Guan LL Steele MA. From pre- to postweaning: Transformation of the young calf’s gastrointestinal tract. J Dairy Sci (2017) 100:5984–95. doi: 10.3168/jds.2016-12474 28527800

[79] He Z Ma Y Chen X Yang S Zhang S Liu S. Temporal changes in fecal unabsorbed carbohydrates relative to perturbations in gut microbiome of neonatal calves: Emerging of diarrhea induced by Extended-Spectrum beta-lactamase-Producing enteroaggregative escherichia coli. Front Microbiol (2022) 13:883090. doi: 10.3389/fmicb.2022.883090 35875583 PMC9301005

[80] Macia L Tan J Vieira AT Leach K Stanley D Luong S. Metabolite-sensing receptors GPR43 and GPR109A facilitate dietary fiber-induced gut homeostasis through regulation of the inflammasome. Nat Commun (2015) 6:6734. doi: 10.1038/ncomms7734 25828455

[81] Schilderink R Verseijden C de Jonge WJ. Dietary inhibitors of histone deacetylases in intestinal immunity and homeostasis. Front Immunol (2013) 4:226. doi: 10.3389/fimmu.2013.00226 23914191 PMC3730085

[82] Wu J Zhou Z Hu Y Dong S. Butyrate-induced GPR41 activation inhibits histone acetylation and cell growth. J Genet Genomics (2012) 39:375–84. doi: 10.1016/j.jgg.2012.05.008 22884094

[83] Singh N Thangaraju M Prasad PD Martin PM Lambert NA Boettger T. Blockade of dendritic cell development by bacterial fermentation products butyrate and propionate through a transporter (Slc5a8)-dependent inhibition of histone deacetylases. J Biol Chem (2010) 285:27601–8. doi: 10.1074/jbc.M110.102947 PMC2934627 20601425

[84] Tao R de Zoeten EF Ozkaynak E Chen C Wang L Porrett PM. Deacetylase inhibition promotes the generation and function of regulatory T cells. Nat Med (2007) 13:1299–307. doi: 10.1038/nm1652 17922010

[85] Thorburn AN McKenzie CI Shen S Stanley D Macia L Mason LJ. Evidence that asthma is a developmental origin disease influenced by maternal diet and bacterial metabolites. Nat Commun (2015) 6:7320. doi: 10.1038/ncomms8320 26102221

[86] Park J Goergen CJ HogenEsch H Kim CH. Chronically elevated levels of Short-Chain fatty acids induce t Cell-Mediated ureteritis and hydronephrosis. J Immunol (2016) 196:2388–400. doi: 10.4049/jimmunol.1502046 PMC4761537 26819206

[87] Husted AS Trauelsen M Rudenko O Hjorth SA Schwartz TW. GPCR-Mediated signaling of metabolites. Cell Metab (2017) 25:777–96. doi: 10.1016/j.cmet.2017.03.008 28380372

[88] Singh N Gurav A Sivaprakasam S Brady E Padia R Shi H. Activation of Gpr109a, receptor for niacin and the commensal metabolite butyrate, suppresses colonic inflammation and carcinogenesis. Immunity (2014) 40:128–39. doi: 10.1016/j.immuni.2013.12.007 PMC4305274 24412617

[89] He Z Ma Y Chen X Liu S Xiao J Wang Y. Protective effects of intestinal gallic acid in neonatal dairy calves against Extended-Spectrum beta-lactamase producing enteroaggregative escherichia coli infection: Modulating intestinal homeostasis and colitis. Front Nutr (2022) 9:864080. doi: 10.3389/fnut.2022.864080 35399688 PMC8988045

